# Collision-enhanced friction of a bouncing ball on a rough vibrating surface

**DOI:** 10.1038/s41598-020-80067-w

**Published:** 2021-01-11

**Authors:** N. D. Smith, M. R. Swift, M. I. Smith

**Affiliations:** School of Physics and Astronomy, University of Nottingham, Nottingham, NG7 2RD UK

**Keywords:** Statistical physics, thermodynamics and nonlinear dynamics, Soft materials

## Abstract

We describe experiments and simulations to investigate the dynamics of a ball bouncing on a rough vibrating surface. Directly measuring the impulse due to each bounce we find that the frictional interaction with the surface is strongly enhanced near to the side wall. The enhanced dissipation arises as a consequence of the coupling between the collision, rotation and surface friction. This dissipation, which for our experimental conditions was estimated to be up to three times larger than the more obvious inelastic collision, can result in an enhanced probability density near boundaries and particle–particle spatial correlations. Our findings imply that the effective particle collision properties cannot be considered independently of the surface’s frictional properties.

## Introduction

There is currently much interest in understanding the statistical properties of systems driven far from equilibrium^1^. Often such systems exhibit complex collective behaviour; examples include jamming in colloids^2^, phase separation in active matter^3^ and pattern formation in granular media^4,5^. A unifying theoretical framework akin to equilibrium thermodynamics^6^ is still lacking for these driven dissipative systems. In thermodynamic equilibrium, mesoscopic modelling relies heavily on the fluctuation-dissipation theorem, which relates macroscopic dissipation to thermal fluctuations^7^. A well-known example is Brownian motion, in which thermal fluctuations are balanced by viscous drag.

Grains vibrated on a roughened surface have some features in common with molecular fluids^8^ while also exhibiting non-equilibrium effects, including anomalous velocity statistics^9–11^ and long-range spatial correlations^12–14^. Vibrated grains, under certain conditions, also show features in common with some active matter systems, such as an increasing probability density as one approaches boundaries^15–17^.

Some common mesoscopic descriptions of driven granular media assume that the interaction between the grains and the surface can be modelled by Gaussian noise^18^ and a velocity dependent drag or frictional term^14,19^. The equations of motion reflect the underlying assumption that the motion of particles can be separated into a spatially uniform term due to particle–surface interactions (noise and friction) and terms due to particle collisions (other particles and boundaries). Such an approximation can be justified as being the leading order terms in a Kramers–Moyal expansion^7^ and is found to be adequate for describing dense granular gases^19^. Factors such as particle rotation are therefore sometimes assumed to be only of secondary importance^20^. In quasi-2d experiments it is also generally true that the rotational degrees of freedom of the particle cannot be explicitly measured.

**Figure 1:**
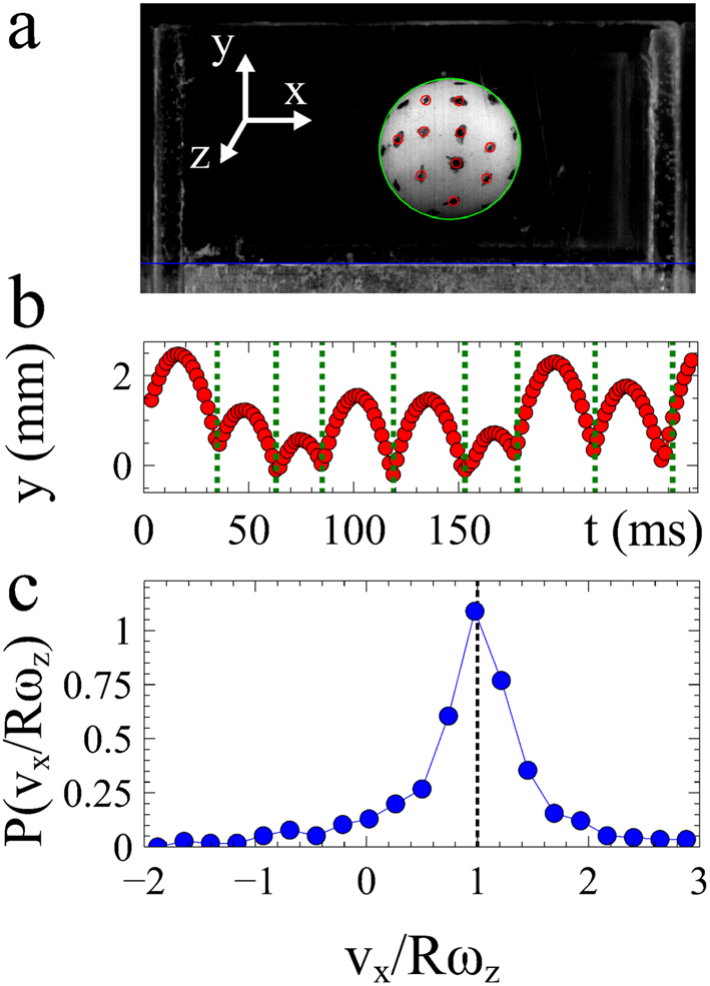
Bouncing ball experiment. (**a**) The position of the surface (blue) and ball (green) are tracked. The rotation of the ball is also measured by tracking the motion of black dots (red) on the ball surface. (**b**) The location of a bounce is identified when ${\mathrm{d}}y/{\mathrm{d}}t$ changes from negative to positive. (**c**) Histogram of $v_x/R\omega _z$. The rolling condition occurs when $v_x/R\omega _z=1$. (Figure prepared using Veusz https://veusz.github.io).

Here we describe experiments using a single sphere vibrated on a roughened surface, confined laterally in a small cell (Fig. 1a). Directly measuring the velocity dependent frictional force that occurs during each bounce of the ball reveals that it depends strongly upon the distance of the particle from the cell walls. Simulations that include particle rotation allow us to explain this behaviour. We find that if rolling and surface friction are combined with a collision (even if elastic) then the frictional loss due to the surface is amplified. This simple mechanism has potential consequences for the density enhancement at boundaries and short range particle–particle correlations. Indeed, we illustrate that this combination of elastic collision, particle rotation and surface friction may play a more influential role than the collisional losses due to inelasticity. This effect could be an important consideration when comparing different experiments and simulations, and in developing mesoscopic models of granular media.

A number of studies have made detailed experimental measurements of the collisional properties of particles impacting surfaces, from which normal and tangential coefficients of restitution can be accurately measured^21,22^. Whilst valuable, in a quasi-2d experiment the bounce of a ball is linked to the preceding bounce(s) and collision(s), which determine the distribution of incident linear and rotational velocities. This is further complicated by the relative phase of the vertically oscillating surface at which bounces on the surface take place^23,24^. The coupling between particle–wall or particle–particle collisions and bounces on the vibrating surface, rather than the basic collisional properties of particles, are the focus of this study.

## Methods

The experiment consists of a small cell (width 30 mm, depth 20 mm), with an aluminium base covered with sand paper (grit size $\sim$ 201 $\upmu$m) and Perspex walls. The cell was vibrated vertically with a sinusoidal motion, amplitude *A*, at a frequency $f=$50Hz, and a range of dimensionless accelerations $\Gamma =A(2\pi f)^2/g$. The motion of a 10 mm diameter Delrin ball was filmed at 500 fps (Optronis CL600x2). For each surface acceleration $\Gamma$, we collected 3 $\times$ 6s movies of the ball’s motion.

For simulations, the ball was modelled as a sphere with both translational and rotational degrees of freedom. It is confined to move and rotate in 2d and bounces on a surface which is created from small particles, spaced at regular intervals. In the simulation the 10 mm diameter ball was confined in the *x* direction by two walls 30 mm apart, as per the experiment. The end walls in the simulation have no loss and any influence of the front and back faces present in the experiment was ignored. The model of the ball has collisional dynamics controlled by a number of parameters: the normal coefficient of restitution *e*, the coefficient of tangential sliding friction $\mu$, the mean radii of the surface particles and the width of their size distribution (full details are given in supplementary information).

The motion, rotation and bounces of the ball, together with the surface motion, were tracked throughout each experiment, Fig. 1a,b (also supplementary information and movie 1). The rotations of the ball were tracked using black ink dots on the ball’s surface. From these measurements we calculated the velocity distributions (supplementary Fig. 2) and the correlation between translational, $v_x$, and rotational, $\omega _z$, motion $v_x/R\omega _z$ (Fig. 1c). For convenience, we define a clockwise rotation of the ball as positive. Using this sign convention, when $v_x/R\omega _z = 1$ the translational motion of the ball matches its rotation as it would when rolling. Whilst this is the most probable value there is a significant distribution, indicating that the ball’s rotation can become out of sync with its translation.

Whilst we can measure the horizontal velocity immediately before ($v_x$) and after ($v^\prime _x$) each bounce, it is not possible to determine the time the ball is in contact with the surface. This is due to both time resolution and the difficulty of defining ‘in contact’ visually. We therefore measure $\Delta v_x = v^\prime _x - v_x$ rather than the force applied to the ball.

**Figure 2:**
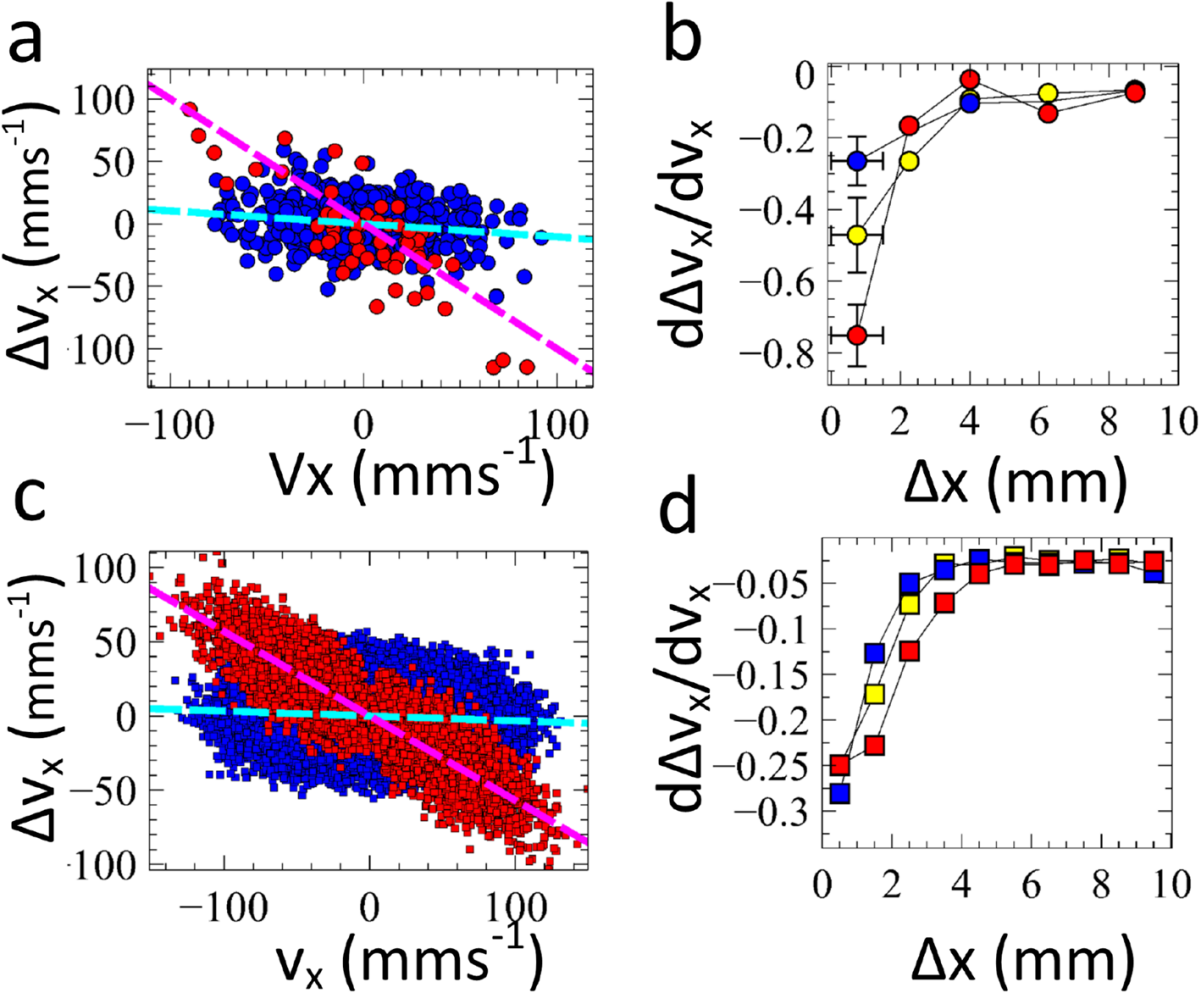
Friction of a bouncing ball. (**a**) Experimental measurement of bouncing ball ($\Gamma$ = 3.25). The change in velocity at each bounce within 1 mm of the end walls (red) and the centre of the cell (blue). The gradient of the line indicates the frictional impulse. (**b**) The frictional impulse at different distances from the side wall. $\Gamma$ = 2.25 (blue), 2.75 (yellow), 3.25 (red). (**c**) Simulation of a bouncing ball. The change in velocity of particles whose last collision was with the end wall (red) and those whose last collision was with the base (blue). (**d**) The frictional impulse for simulation at different distances from the side wall. $\Gamma$ = 2.25 (blue), 2.75 (yellow), 3.25 (red). (Figure prepared using Veusz https://veusz.github.io).

## Results and discussion

Figure 2a shows an example dataset for a ball bouncing on a surface with dimensionless acceleration $\Gamma$ of 3.25. For any given horizontal velocity, $v_x$, there are a large range of possible changes in velocity, $\Delta v_x$. However, as the horizontal velocity of the ball prior to a bounce becomes increasingly positive, the change in velocity is on average increasingly negative. The gradient of a linear fit to ${\mathrm{d}}\Delta v_x/{\mathrm{d}} v_x$ characterises the average fractional change, which is proportional to the frictional impulse. The blue dots shown in Fig. 2a occur when the ball is in the central region of the cell. In all experiments we observed a negative gradient ${\mathrm{d}}\Delta v_x/{\mathrm{d}} v_x \approx -0.08$. However, separating out the data that is obtained when the ball is within 1 mm of the two end walls resulted in a much larger negative gradient $\sim -1$. Indeed Fig. 2b, which shows the bounce data binned by distance from each end wall, indicates that the gradient is substantially affected a few millimetres from the end wall. It is well known that there are collisional losses due to boundaries that introduce spatial variations in the velocity distribution^15^. However, we emphasise that the behaviour reported here is very different. In these experiments the velocities are extracted immediately before and after each bounce on the horizontal surface. The measured value of $\Delta v_x$ is not therefore due to collisional losses with the end walls.

Whilst this finding is intriguing, there are a number of inherent limitations with the experiment. Firstly, the strong confinement of the particle in the *z* direction means part of the measured frictional interaction may arise from the ball glancing off the front or rear faces of the cell^20^. Secondly, the ball’s velocity or angular velocity vector may not be always solely in x or z. We would therefore measure a reduced velocity component $v_x$ when the ball moves in the z direction (see discussion supplementary information). Thirdly, it is possible that collisions with the moving sidewall could modify the distribution of vertical velocities relative to the surface. Since the relative impact velocity sets the normal contact force it could modify the friction. It is not clear that either of the first two mechanisms should correlate with the *x* position of the ball and measurements of the relative vertical velocity of the ball showed no significant changes near to the sidewalls. However, in order to understand the enhanced friction and rule out such experimental contributions, we performed simulations of the bouncing ball^25,26^.

Our simulation exhibits a similar behaviour to the experiment with a substantial increase in frictional loss associated with the wall. The simulation allows us to directly detect the collision with the wall and hence in Fig. 2c the red points indicate the bounce immediately following a collision with the wall, thereby enabling a much more direct measurement of the effect of the wall than is possible in experiment. Figure 2d shows how the mean frictional loss upon bouncing varies with position in the cell. There are quantitative differences between simulation and experiment, presumably due to the differences outlined earlier or the simplistic assumption that the sandpaper surface can be modelled as a set of spheres. However, it appears that the 2d simulations capture the essential features of the experiment and exhibit a similar spatial dependence on the distance to the end wall. This is encouraging since although a number of parameters were tuned to achieve the correct ball dynamics in the centre of the cell, the changes due to the wall arise naturally with no additional constraints.

**Figure 3:**
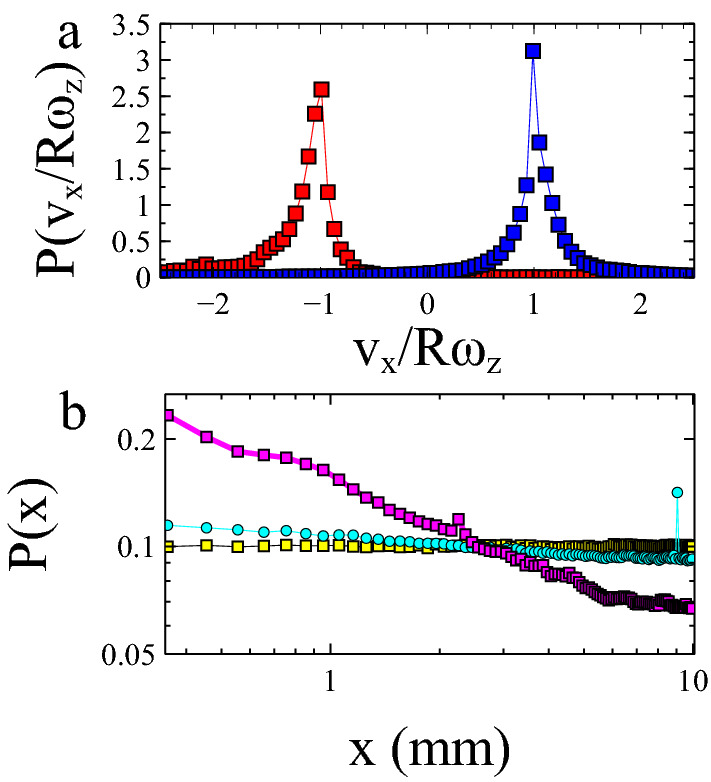
The effect of the wall. (**a**) Histograms of $v_x/R\omega _z$ for a simulated bouncing ball immediately after (red) or without (blue) a preceding collision with a wall. (**b**) Probability density plot as a function of the position in the cell on a log–log scale. The magenta plot shows the case of a rolling ball which undergoes an elastic collision with the cell walls. Both the remaining plots have zero tangential friction coefficient which stops the ball rotating. These simulations are done with an elastic (yellow) and inelastic (cyan) wall collision. Rolling results in a much larger density enhancement at the wall than inelasticity. The density profile shows a power law like dependence. (Figure prepared using Veusz https://veusz.github.io).

To understand the origin of the enhanced loss near the walls, one *must* include the rotational motion of the ball. Consider an idealised case of a ball which bounces across a flat stationary surface with a tangential friction coefficient. The ball will experience no frictional losses provided its translational velocity ($v_x$) equals its rotational velocity ($\omega _z$) multiplied by the ball radius (*R*) (i.e. $v_x/R\omega _z = 1$). This is because the relative contact velocity between ball and surface at the point of contact ($v_c= v_x - R\omega _z$) is zero. This means the ball and surface do not slide past one another, generating friction, but roll during the bounce. If $v_x/R\omega _z$ deviates from 1 the ball experiences a frictional force which either increases $v_x$ and decreases $R\omega _z$, or vice versa, to push the ball back towards the condition for rolling. In the full simulation, in the middle of the cell these deviations in $v_x/R\omega _z$ from rolling occur due to the random kicks received from the roughened base. One therefore observes a kind of dynamic equilibrium in which the kicks result in deviations from $v_x/R\omega _z = 1$ (Figs. 1c and 3a) but the nature of a ball bouncing on a frictional surface is to tend back towards the condition for rolling.

However, upon reaching the wall there is an additional factor to consider. The collision of the ball with a smooth wall reverses the horizontal velocity ($v^{\prime }_x=- v_x$) of the ball without changing the sign of its rotational velocity ($\omega ^{\prime }_z= \omega _z$). If the ball prior to the collision satisfies the rolling condition $v_x=R\omega _z$, this would then be modified to a value of $v^\prime _x/R\omega ^\prime _z \approx -1$. Figure 3 illustrates how the reflection due to a wall results in a complete reversal of the distribution of $v_x/R\omega _z$. The data in red includes only those points which had hit the side wall prior to bouncing. Whilst the magnitude of the frictional force is $\upmu N$, where *N* the normal force due to contacts, the sign of the frictional force is determined by the relative contact velocity of the point of contact. In this case $v_c = v^\prime _x - (R\omega ^\prime _z) = -2v_x$. Consequently, the average behaviour results in a large negative frictional impulse.

The introduction of frictional/lossy side walls, as might occur in the experiment, undoubtedly complicates this picture but the change in rotation has no a priori reason to be correlated with the reversal of the translational velocity. Consequently, when a bounce occurs immediately after a collision with the wall it results in a larger subsequent frictional interaction with the surface, which would explain the strong enhancement in measured loss in Fig. 2b,d near to the wall. This apparently simple loss mechanism which we refer to as relative contact velocity friction has not, to our knowledge, been strongly highlighted in the literature but could have important consequences for understanding granular systems.

Our result implies that there is an observed loss due to the wall (even if the collision is elastic) which varies with the frictional nature of the vibrating base. In this simplest of non-equilibrium experiments, losses due to a perfectly elastic wall result in an increased probability density near to the boundary (see Fig. 3b). That this is a consequence of the rotational nature of the particle can be seen through comparison with a system where the tangential friction coefficient is set to zero removing the rotational torques on the ball (yellow). In such a situation the probability density is completely uniform. It is well known that active matter systems result in density enhancements at a wall due to a “memory” or persistence^27^. Similar effects arise here, where a “memory” of the previous bounce and direction of travel arise as a consequence of the persistence of rotation during a collision with boundaries.

The apparent energy loss of a particle when it collides with the side wall can therefore have two contributions: the inelasticity of the collision and the increased friction during the next bounce on the surface. An interesting question is: which of these mechanisms is more pronounced? The answer to this question clearly depends on the details of the experiment and relative strengths of, for example, the inelasticity of the collision and amount of friction. However, we can show that, at least in our experiment, the coupling between elastic collision, rotation and friction is much more significant. Figure 3b shows how the probability density of the particle in the cell depends on distance from the wall. The losses of the rotating ball, colliding with an elastic wall can be compared with a non-rotating ball colliding with inelastic side wall (r=0.8). The probability density enhancement is much less pronounced due to inelasticity. Density enhancements near walls have been studied for a variety of active matter systems. Non-interacting Active Brownian Particles were shown to give rise to an exponential density enhancement at the wall^16^. Similar effects have also been observed for motile bacteria^17^, though in this latter case the enhancements are a collective phenomenon. Our data with rotation appears to result in a power law dependence of the probability density with distance (see Fig. 3b, inset), with a slope of approximately -0.4. It is not clear exactly what controls the exponent in each of our simulations, though the addition of rotation appears to increase the value significantly compared to the inelastic case. Power-law correlations are also know to exist in one-dimensional particle models without rotation^18,28^.

Many experiments focus on regions away from the boundaries to minimise their influence, and simulations frequently use periodic boundary conditions. However, it should be realised that the relative contact velocity friction loss mechanism is also relevant to inter-particle collisions. If one considers two particles which satisfy the rolling condition undergoing a collision, the relative particle velocities would also reverse, without a commensurate reversal in angular velocity. Thus the frictional interaction between a particle and the base should also enhance loss in particle–particle collisions and result in post collisional density correlations.

**Figure 4:**
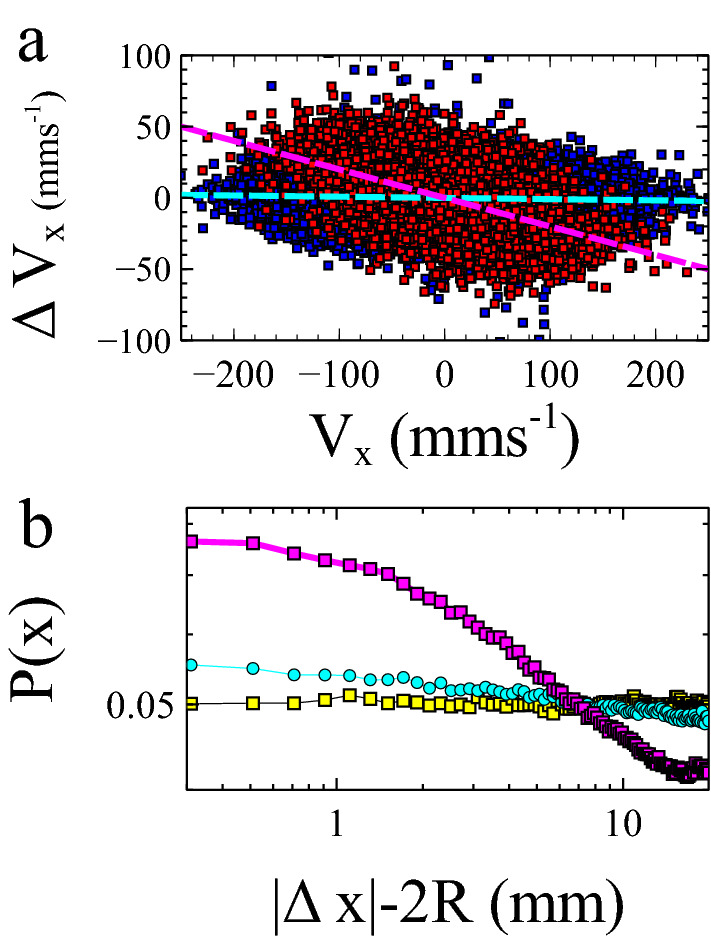
Simulations of 2 bouncing balls in a 60 mm wide periodic box. (**a**) frictional impulse due to a normal bounce (blue) and a bounce following a collision with the other ball (red). (**b**) Separation probability between the two particles on a log–log scale. We compare elastic collisions with (magenta) and without (yellow) rolling, and an inelastic collision without rolling (cyan). The relative contact velocity friction mechanism provides the most significant loss mechanism. (Figure prepared using Veusz https://veusz.github.io).

To demonstrate this we performed simulations of two 10 mm diameter balls bouncing on a vibrated surface as before. The cell has twice the width of our earlier simulations (60 mm) and has periodic boundary conditions as opposed to hard walls. We consider the simplest possible case in which the spheres have a normal coefficient of restitution and tangential loss coefficient when interacting with the vibrating surface. We consider the cases in which particle–particle collisions are either elastic or inelastic $(e=0.8)$. The changes in velocity as a function of initial velocity at each bounce of one of the balls are shown in Fig. 4a (compare Fig. 2c). We compare those changes immediately following a collision (red) with all other bounces (blue). The mean frictional impulse received after a collision is significantly enhanced for a given horizontal velocity. In Fig. 4b we compare the probability density of finding particles at a given separation (compare Fig. 3b). The quantity $|\Delta x| - 2R=0$ when the two balls touch. As before, in the absence of a tangential friction component (no rolling), the ball’s probability density does not depend on position (yellow). The addition of inelastic collisions (cyan) results in a small correlation in the two particle’s positions. However, the addition of relative contact velocity friction (with elastic collisions) leads to an enhanced probability density of finding particles close together (magenta). Both these results are qualitatively the same as those observed for ball–wall collisions, illustrating that these ideas need to be taken into account even in large systems where the effects of the wall can be ignored. Such behaviour is similar to that observed in active matter systems in which the persistence of motion can result in motility induced clustering^3^. This raises interesting questions about to what extent driven granulars and active matter systems share an underlying physics^27^.

Relative contact velocity friction represents an additional dissipative mechanism that leads to collective behaviour in granular systems. Experimentalists routinely report the size and material of granular particles used in an experiment in a bid to allow comparisons with other work to be made. However, our study shows that this information, without knowledge of the surface used, leads to an effective uncertainty on the basic collisional properties of particles, thereby severely hampering reproducibility or a quantitative comparison. This may contribute to an explanation for why apparently subtle differences in granular experiments have frequently lead to qualitatively different behaviours^29^.

**Figure 5:**
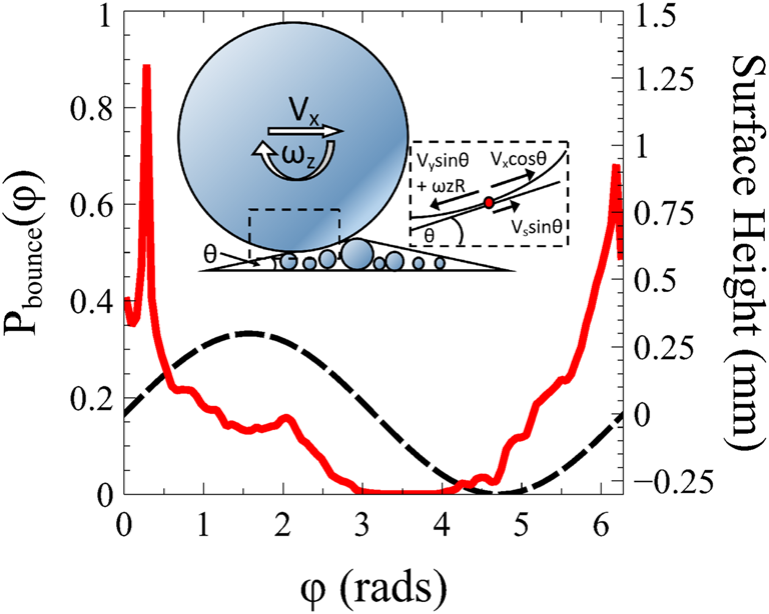
Histogram of the phase of ball bounces (left) relative to the surface height (right). The probability of a collision during the upwards phase of the surface is strongly enhanced. Inset: Schematic showing the asymmetry due to surface structure. As $v_x$ increases, the relative probability of hitting ‘upwards’ sloping collections of particles increases. The contributions to the relative contact velocity of the ball–surface contact when components are taken parallel to the local surface geometry. (Figure prepared using Microsoft Publisher and Veusz https://veusz.github.io).

Our argument is that if a collision results in the relative velocity of the contact point $v_c$ at the next bounce being in the opposite direction to $v_x$ there will on average be a reduction in the particle velocity due to the tangential sliding friction. Indeed we now proceed to show how the same mechanism is partially responsible for the regular frictional interactions of the ball with the vibrating base itself. There are two asymmetries during a bounce, which on average produce a backwards relative motion of the contact point, resulting in an enhanced frictional force at the next bounce.

Firstly, there is an asymmetry due to the relative motion of the surface and ball. Figure 4 shows a histogram of the phase of ball bounces relative to the surface oscillation. The ball mostly collides with the upwards moving surface.

The second asymmetry arises from surface geometry, combined with the horizontal velocity of the ball. The roughened surface consists of particles of different sizes arranged randomly next to one another. The probability of hitting an upward sloping collection of particles relative to downward sloping collection of particles increases with $v_x$ (see schematic Fig. 5, inset). Since the coefficient of restitution modifies the velocity normal to the surface, and the average local surface normal has a horizontal component that opposes $v_x$, the ball will tend to slow down (cf rolling friction)^30^. However, in addition to this collisional loss, there is a frictional mechanism analogous to that outlined above in connection with the wall collision. Assuming, on average, a positive surface velocity $v_s$, a negative vertical ball velocity $v_y$ and a positive local surface slope $\theta \sim a/R$, an approximate expression for $v_c$ is

1 $$\begin{aligned} v_c \approx v_x-\omega _z R-(v_s - v_y)\frac{a}{R}. \end{aligned}$$

It is the third term on the RHS of this equation, which is on average negative, that breaks the symmetry of the contact velocity. The frictional force which on average acts forwards in this case applies a ‘backspin’ to the ball which is *larger* than experienced on a static surface. This reduction in rotation relative to translation then results in enhanced friction when the ball next bounces. As a test of these ideas we have compared simulations with and without a tangential loss coefficient $(\mu =0)$. Without Collisional losses, as experienced in rolling friction, the result is a measured value of $d\Delta v_x/ d v_x$ about a factor of 3 smaller than in the case with relative contact velocity friction present.

## Conclusions

In conclusion, the rotational motion of a ball on a frictional surface has important consequences for the collisions between particles and experimental boundaries. Collisions result in changes to the horizontal velocity without commensurate changes in the rotation, resulting in a strongly enhanced frictional loss. This implies that the ball’s apparent loss during a collision can depend more on the increased frictional interaction with the surface than the inelasticity of the wall, or the particle–particle collisions. It was also shown that two asymmetries which arise from the phase of the ball bounce relative to the surface motion and the local surface slope lead to a similar mechanism, which is partially responsible for surface friction. This has implications for mesoscopic modelling approaches but also for the comparison of different experiments^9,10,29,31^. The behaviour also highlights a deeper connection between shaken granular and active matter systems^27^.

## Supplementary Information

Supplementary Information.

Supplementary Video.
