# Supplementary material for: Collision-enhanced friction of a bouncing ball on a rough vibrating surface
Source: Sci Rep. 2021 Jan 11;11:442. doi: 10.1038/s41598-020-80067-w (PMC7801373; doi:10.1038/s41598-020-80067-w)
Supplement: Supplementary file 1 — Supplementary Information. [file 41598_2020_80067_MOESM1_ESM.pdf]

Collision-enhanced friction of a bouncing ball on a rough vibrating surface.

Supplementary Information – N.D. Smith, M.R. Swift, M.I. Smith

Supplementary Video 1

The supplementary video shows a small section of a video of a ball bouncing on a P80 surface with vertical dimensionless acceleration $\Gamma \sim 3.25$ and freq=50Hz. The tracking is indicated by the various different coloured annotations. The top of the aluminium surface is tracked (blue line). The true height is shifted slightly upwards by the presence of the wet and dry. The offset required is ascertained visually and the same value is then used throughout all the videos. The centre of the ball is found from a least squares fit of a circle to the ball outline (green). The rotations of the ball are found from tracking the motion of small black dots placed on the ball surface (ringed in red). Tracking the motion of these dots relative to the ball centre allows rotations to be measured.

Experiment Particle Tracking

To analyse the motion of the ball we used a least squares fit of a circle to the contour points associated with the ball edge. The rotations of the ball were also measured by tracking the motion, relative to the ball centre, of small pen marks placed on the ball (see figure 1a and supplementary movie 1). Since the dots are located on the surface of the ball, the relative x, y *and* z locations can be measured or calculated. This enabled us to measure the distributions of the x component of the ball's velocity and the z component of its angular velocity (see supplementary figure 1) for different surface accelerations. Tracking the motion of the ball over time, each bounce was detected by the change in sign of the vertical velocity from negative to positive (see figure 1b).

Since the motion of the ball is not always in the x-y plane, as in the simulation, this procedure may introduce some differences between the measured distributions of Vx/Rωz. We believe these differences are reduced by several factors:

1. The small depth dimension of the cell appears to encourage more lateral than front to back motion. Hence rotation is often relatively close to being about the z axis.

2. The component of the velocity and angular velocity vectors onto the x and z axis respectively both depend on cosθ. The quantity $V_x/R\omega_z$ thus results in this angular dependence being cancelled out.

The distributions in figure 1c and 3 are in relatively close agreement but the 3D ball will likely result in an increased probability of small values of vx, relative to the simulation, since it can roll in the z direction.

Simulation details

We simulate the translational and rotational dynamics of the ball, confined to move in a two dimensional vertical plane. Collisions between the ball and the base were modelled using a linear spring dashpot model [Hermann 1998]. Tangential forces were modelled using a sliding coefficient of friction with a small velocity cutoff [Stewart 2000]. The normal coefficient of restitution was estimated directly from experiment ($e \sim 0.5$). Analysing the relative vertical velocity v_y and v'_y of the ball and plate we matched this to the simulation by measuring the mean loss per bounce. The mean radii of the surface particles was set equal to the reported grit size of the sandpaper. The width of the particle size distribution and tangential loss coefficients were adjusted by comparing the velocity and angular velocity distribution of the experiment and simulation for *one* particular surface acceleration (see supplementary figure 2 below). Keeping all these parameters constant the surface acceleration Γ in simulation was then varied and a comparison was made with the linear and angular velocity distributions measured in the corresponding experiments. Good agreement between experiment and simulation was achieved over the range of surface accelerations investigated (2.25-3.25).

Care was also taken to ensure that the time-step used in the simulations, and the value of the cut-off, did not significantly influence the measured behaviour.

Chattering during simulations

In the experiment the definition of a bounce is simply defined by the velocity before and after the ball visually leaves the surface. In contrast, the much finer timer resolution

accessible via the simulation meant that occasionally one observes the ball being caught by the descending surface and undergoing chattering like modes; that is large numbers of infinitesimally small bounces. These modes have been extensively studied in the context of the bouncing ball as a model chaotic systems [Luck 1993]. In practise these modes result in the ball being captured by the surface and then relaunched during the upward motion of the surface where the surface acceleration becomes less than –g. Since our interest is in the macroscopically observable bounces exhibited in the experiment we filter these bounces from the simulation data based on the time between bounces. Failure to do this distorts the data, since a “single” visually observed bounce would appear to be a vast number of changes in velocity.

Comparison of velocity and angular velocity distributions

In order to ensure robustness in our simulation results, for each set of experimental parameters we created 50 different randomly generated surfaces. In analysing the results for the same experimental parameters, but from different surface realisations, we discovered that most surfaces resulted in an approximately Gaussian velocity distribution with similar mean and variance. There were however a minority of surfaces which resulted in a strongly spiked distribution at zero velocity (See figure S1).

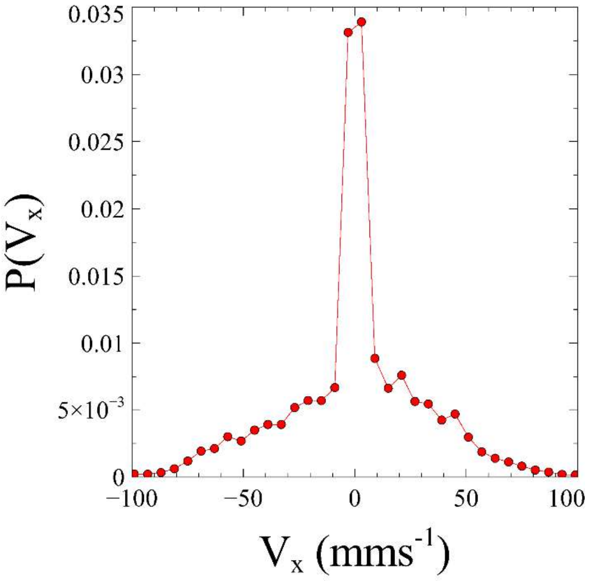

S1) Atypical velocity distribution observed for a minority of surface realisations. Due to the 2d nature of the surface particles can get trapped at particular locations, distorting the velocity distribution.

It appears that by chance one can generate small regions of a surface in which the ball can become trapped at a single position. The ball will then bounce for very long times at a single position. We believe this effect is amplified by the 2D nature of the simulation since in the experiment any natural "hollows" would only confine the ball in the x-direction and the ball could easily escape by travelling a small distance in z. To prevent this unduly influencing our results we excluded strongly spiked velocity distributions which were easily observable from the strong reduction in the mean speed.

With this caveat figure S2 shows that the simulation nicely captures the velocity and angular velocity distributions measured in the experiment.

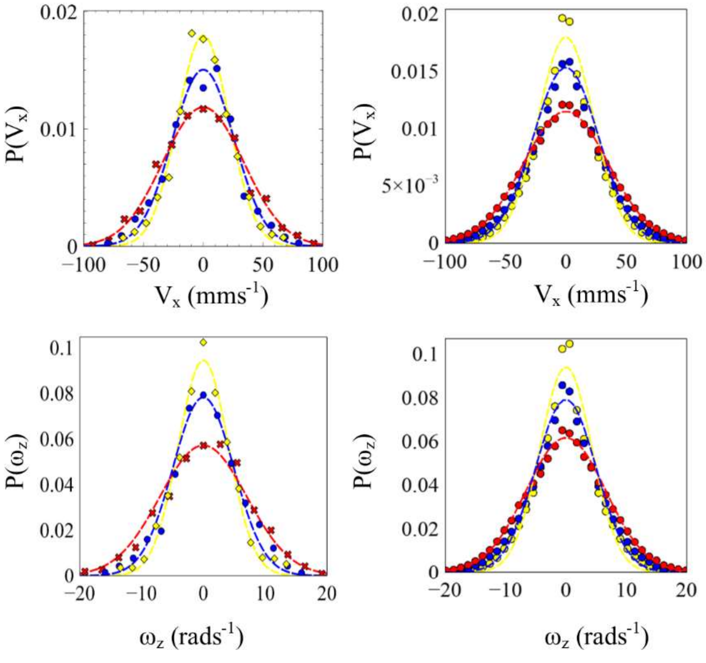

S2) A comparison of the measured velocity (top row) and angular velocity (bottom row) distributions for both experiment (left column) and simulation (right column).
